# Decolorization of Palm Oil Mill Effluent by *Klebsiella Pneumonia* ABZ11: Remediation Efficacy and Statistical Optimization of Treatment Conditions

**DOI:** 10.3389/fmicb.2020.00675

**Published:** 2020-05-13

**Authors:** Mohammed Abdulsalam, Hasfalina Che Man, Zurina Zainal Abidin, Khairul Faezah Yunos, Aida Isma Idris

**Affiliations:** ^1^Department of Biological and Agricultural Engineering, Faculty of Engineering, Universiti Putra Malaysia, Serdang, Malaysia; ^2^Department of Agricultural and Bio-Resources Engineering, Faculty of Engineering, Ahmadu Bello University, Zaria, Nigeria; ^3^Department of Chemical and Environmental Engineering, Faculty of Engineering, Universiti Putra Malaysia, Serdang, Malaysia; ^4^Department of Food and Process Engineering, Faculty of Engineering, Universiti Putra Malaysia, Serdang, Malaysia; ^5^Department of Chemical Engineering, Segi University, Kota Damansara, Malaysia

**Keywords:** POME, decolorization, colorants, *Klebsiella pneumonia* ABZ11, kinetic-model, optimization

## Abstract

Colorants contained in palm oil mill effluent (POME) are recalcitrant and carcinogenic in nature. The commonly applied ponding treatment methods have been reported inefficient for remediating the concentration of the colorants before discharge. The need for sustainable and efficient treatment technique is crucial in order to preserve the environment. In this view, this study reported the first attempt to decolorize POME using a proliferate *Klebsiella Pneumonia* ABZ11 at varied inoculum sizes of 5–25% (v/v), initial color concentration (650–2,600 ADMI) and treatment time of 5-40 h. The treatment conditions were optimized using Response Surface Methodology. At optimal conditions of 20% (v/v) inoculum size, initial-color concentration of 2,600 ADMI, initial pH of 7 and 35 h treatment retention time, over 80.40% color removal was achieved with insignificant disparity compared with the model predicted value of 81.538%. Also, the Monod model excellently described the decolorization kinetic process with 0.9214 coefficient of correlation (*R*^2^), and the calculated maximum growth μ_*max*_) and half-saturation constant (*K*_*s*_) were 7.023 d^–1^ and 340.569 ADMI d^–1^, respectively. This study revealed that the *Klebsiella Pneumonia* ABZ11 was highly prolific and such feature may favor a synergistic biodegradation process.

## Introduction

Industrial processing and extraction of oil palm are usually associated with the colossal generation of colored wastewater commonly known as palm oil mill effluent (POME). Reports have shown that about 2.5 t of raw colored POME are generated from every ton of crude oil-palm extracted (Choong et al., 2018). The dark brownish color of the effluent was due to excessive concentration of tannins, melanoidin and lignin compounds (Tamrin and Zahrim, 2017; Tan et al., 2017). However, reductase activities of microbes on the complex colorants could result in the formation of aromatic ring amines compound which is more toxic than the precursor compounds (Neoh et al., 2013). Although, the initial form of the colorants compounds may not be considered as cytotoxic or carcinogenic substances. Conversely, decomposition of these compounds after discharged into the environment could lead to the formation of aromatic amines which are capable of inducing cancer or tumors (Ganapathy et al., 2019). More so, continues accumulation of POME in waterways is responsible for diminishing photosynthetic processes of the planktons, thereby distorting the stable ecosystem in the aquatic environment (Abdulsalam et al., 2018b). Therefore, the need for effectual treatments technique to reduce the concentration of the colorant before discharge into the environment is critical.

However, ponding system has being the most common method used for treating POME before discharge into the waterways but it has been proven ineffectual, particularly for color removal (Rana et al., 2017; Iskandar et al., 2018). Though physical and/or chemical treatment methods such as adsorption, chemical oxidation and reduction, precipitation, chemical-photolysis, and electrochemical processes decolorizes POME (Abdulsalam et al., 2018a), but they are economically unsustainable and often resulted in the generation of acidic secondary effluent which requires further treatment before discharge. Nonetheless, remediation of organic contaminants in POME using microorganisms such as fungus and bacteria, had demonstrated a promising performance. A consortium of fungi which constitutes *Yarrowia lipolytica, Trichoderma viride* spores, *Saccharomyces cerevisiae*, and *Trichoderma viride* mycelium reduces the initial color concentration by 60–70% under anaerobic condition (Bala et al., 2018). Also, the Brown-rot and White-rot fungus are widely applied for degradation of lignocellulose compounds in POME (Hermosilla et al., 2018). On the other hand, fungus mostly exhibits poor adaptability due to fluctuation in environmental factors such as pH, temperature and oxygen compositions, alongside with difficulty in genetic modification (Sharma et al., 2018).

As a result of the malleability and potency properties of bacteria, its application for wastewater treatment has gained significant attention (Yang et al., 2018; Fu et al., 2019). For example, it performed excellently in reducing the activity of xylanase in POME (Prasertsan et al., 2017), and production of cellulose, lipase, protease and exoglucanase (de Souza et al., 2015; Baraniya et al., 2016; Louhasakul et al., 2016). In another study, Ohimain and Izah (2017) applied an indigenous *Penicillium* consortium on different sources of POME sludge to produce lignin peroxidase. At pH of 7.3, it was observed that the *Penicillium* sequestered from fruit bunches demonstrated outstanding performance in terms of biodegradation of the contaminants present in the POME. Bala et al. (2018) isolated bacteria consortium from POME and applied for contaminants remediation. From the results, they concluded that the community microorganisms have a synergistic removal efficiency of 90.23, 91.06 and 92.23% for BOD, COD and TSS, respectively. Also, Cheah et al. (2018) used POME as a culture media for the cultivation of biomass using the combined *Pseudomonas* sp. and microalgae. The combined bacteria-algae give higher biomass yield (185.7 mg/L/day) and better pollutants degradation. Despite all these efforts, bio-decolorization of POME remains a serious challenge, as the existing techniques were unable to certify the discharge standard limits for color (Lee et al., 2019).

However, the applications of *Klebsiella Pneumonia* ABZ11 for POME decolorization under anaerobic conditions have received little or no attention despite its unique prolificacy and rapid adaptability to change in environmental factors. More interestingly, *Klebsiella Pneumonia* ABZ11 has been characterized to exhibit xenobiotic metabolism on polyphenol and lignocellulose substances using secreted hepatic extracellular polymeric substances and enzymes to facilitate oxidation, reduction, hydrolysis and/or hydration (Xu Z. et al., 2018). This implies that at favorable conditions, the bacteria could swiftly decompose the bio-polymeric colorants (such as the phenol, lignin, tannin and melanoidin) contained in POME (Xu R. et al., 2018). These are the main reasons that motivated this study in order to bridge the existing dearth information on the application of *Klebsiella Pneumonia* ABZ11 for POME decolorization. In this view, the inoculum was cultured to activate the stock, and then the viability was analyzed using optical density technique. Afterward, decolorization performance was examined at varied inoculum sizes (5–25% v/v), initial color concentrations (650–2,600 ADMI), and treatment retention time (5–40 h), while the initial pH of 7 and 120 rpm agitation speed remained fixed throughout the experiment. The treatment conditions were optimized using response surface methodology and the regression model was validated. The accuracy of the prediction using the model was examined based on the coefficient of correlation (*R*^2^) and normality analysis. In addition, the results obtained were statistically analyzed using Design-Expert 10.0.7 and the significance of the treatment factors were examined based on the magnitude of the *F*-values, while the *P* value less than 0.05 is considered to be statistically significant with 95% confidence level.

## Materials and Methods

### Experimental Materials

Nutrient broth (MERCK 1.05443.0500); Nutrient agar (MERCK 1.05450.0500); Micropipette tips (1,000 and 100 μL); microcentrifuge tube (1 mL); wire loops; disposable petri dish and Erlenmeyer flasks (250 mL) were all procured from SIGMA ADRICH (Merck). Also, about 25 L of final discharged POME was collected from an Oil Palm Milling industry, located at Carey Island, Malaysia The visible debris in the sample was separated using a cotton-filter and the initial physicochemical properties were determined using a standard procedure (APHA, 2005). Then, the remaining bulk of the filtrate sample was stored in a chiller at 4°C prior to the further use. In addition, both solid and liquid culturing media were applied in this study. The solid media was prepared using nutrients agar and petri dish following the standard procedure as stipulated by the manufacturer (SIGMA ADRICH). The nutrient agar is composed of partially digested protein (5 g/L); sodium chloride (8 g/L); beef extract (3 g/L) and agar (15 g/L). Approximately 20 g of the nutrient agar was dissolved in 1,000 mL of distilled water under continues stirring condition at 90 rpm and 60°C and then autoclaved at 121°C temperature for a duration of 15 min. Afterward, 100 μL of the homogenous nutrient solution was pipetted into the agar plates (petri dish) and allowed to dehydrate under the laminar flow cabinet. Similar procedures were employed during the preparation of liquid media. However, only 8 g of the medium (compositions: yeast extract-2 g/L, beef extract-1 g/L, NaCl-5 g/L and peptone-5 g/L) was dissolved in 1,000 mL of distilled water, stirred thoroughly at steady agitation of 120 rpm. The mixture was autoclaved at 121°C for 15 min and then stored at 4°C.

### Microorganism

The isolated *Klebsiella Pneumonia* ABZ11 from Antarctica seawater applied in this study was obtained from Universiti Teknologi Malaysia. The bacterium was analyzed using 16S rDNA array and the sequence shows a 99% correlation to *Klebsiella pneumoniae* with Accession Number of KX266892 (Mohammed and Zaharah, 2019)^1^. The 16S rRNA gene PCR amplifications were conducted using universal primers, such that the Forward primer and Reverse primer reads 14-F: 5′-AGAGTTTGATCCTGGCTCAG-3′ and 1492-R: 5′-CGGTTACCTTGTTACGACTT-3′, respectively. The detail reports on the screening, identification including the phylogenetic tree constructed have been reported and published previously (Mohammed and Zaharah, 2019). More so, additional information on the genomic DNA, PCR amplification, partial gene sequence and the phylogenetic relationship of Klebsiella sp. of the ABZ11 were available as Supplementary Figures S1–S4, respectively.

### Preparation of Inoculum and Growth Analysis Using POME as a Media

A single colony was picked into 15 ml of nutrient broth (liquid media) contained in a tube. Initially, the media was adjusted to a pH of ≈7 using 0.1 M of HCl solution. Afterward, the inoculated media was incubated at 35°C and 120 rpm agitation for only 16 h (Singh et al., 2013). The activated inoculum was transferred into a conical flask (500 ml capacity) containing 85 mL of POME solution (25% color concentration i.e., DF = 4), then incubated for 72 h at 35°C and steady agitation of 120 rpm. At an interval of 4 h, about 2 mL of the sample was taken and used for the growth analyzed until the end of the incubation period (72 h). The analysis includes optical density reading (OD), coliform count (CC) and maximum growth analysis (μ_max_) (based on Monod Kinetic model).

#### Optical Density

The OD was monitored by analyzing the changes in the turbidity of the incubated sample using UV-spectrophotometer (HACH 4000U) at a preset absorbance wavelength of 600 nm. Initially, 1 mL of the incubated samples were centrifuged at 10,000 × *g*, for 15 min. Supernatant of the centrifuged samples was discarded, while the accumulated cell pellets were suspended in brine solution of 1% concentration. Then, the suspended cell pellets were placed in a sterilized vial tube for the OD reading at 600 nm.

#### Coliform Count

The colonies counts were determined in separate experiments using serial dilutions technique in a brine solution of 1% concentration with an initial stock of 1.0 ml. Each diluted stock was agitated to ensure a homogenous mixture. Afterward, the content was plated by pipetting 1 mL into the sets of nutrient agar media and then cultured at a temperature of 35°C for 24 h. The grown culture was used to determine the number of colonies formed (CFU/mL) with respect to the corresponding dilution factor, (DF: 10^1^ to 10^10^) using Eq. 1.

(1) $$\mathrm{CFU}/\mathrm{ml}=\text{DF}\times N_{t}$$

where; DF is the dilution factor; *N* is the number of colonies formed.

#### Growth Kinetics

The maximum growth (μ_*max*_) were determined using the Monod Kinetic Model. For the purpose of this study, only the lag and exponential phase of the bacteria growth were considered in the model equations, since the maximum growth of the microbes was defined based on retention time (Park et al., 2015). Thus, only the substrate utilization data obtained in the first 20 h of treatment time were fitted into the kinetic model. This procedure will reflect the more accurate result of the maximum growth during the bio-decolorization process with minimal bias (Park et al., 2015). The general form of the Monod Kinetic Model is expressed in Eq. 3, which rearranged to obtain Eq. 4;

(2) $$\frac{1}{R\,T}=\frac{\mu_{\text{max}}\times C_{e}}{K_{s}+C_{e}}$$

(3) $$R\,T=\left(\frac{K_{s}}{\mu_{\max}}\right)\,\frac{1}{C_{e}}+\frac{1}{\mu_{\max}}$$

where; *RT* is the treatment time (h) and *C*_*e*_ is the colorant concentration of the treated sample (ADMI). The maximum growth (μ_*max*_) and the half-saturation constant (*K*_*s*_) were obtained from the gradient and interception of the regression curve of 1/*S*_*e*_ with respect to *RT*.

### Decolorization Efficacy Using Batch Experimental Approach

**FIGURE 1:**
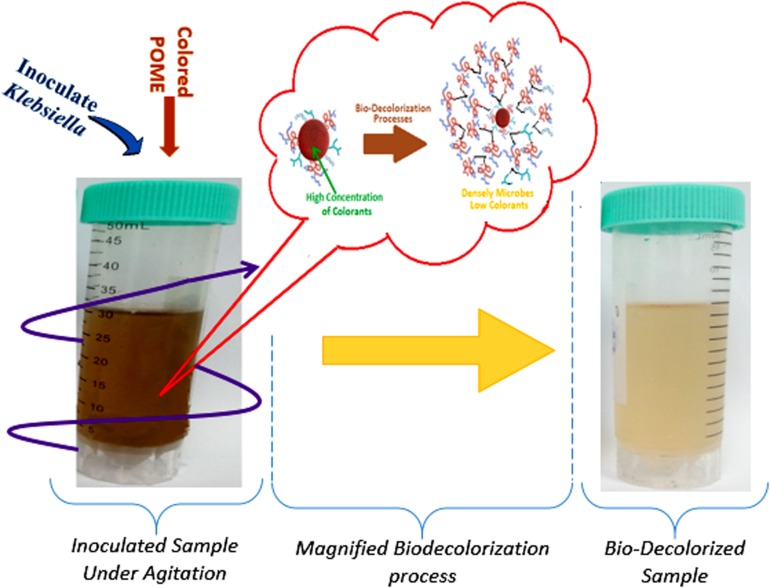
Schematic graphical abstract of decolorization batch experiment at a steady agitation.

The batch decolorization experimental layout was developed using randomized multilevel categoric design (Design Expert Version-10.0.7) with a total of 120 runs and a fixed initial pH of ≈7 was maintained throughout the experiment. The schematic representation of the batch experimental set-up is shown in Figure 1. The cultured *Klebsiella Pneumonia* ABZ11 in the liquid media was used as inoculum at varied sizes (5–25% v/v) to examine its decolorization performance in a prepared POME sample of varying color concentrations (25–100%) and retention time (5–40 h). it should be noted that the color concentrations were varied by adding distilled water at dilutions factors of 4, 3, 2, and 1, and these give a corresponding color concentration of 650, 1,300, 1950, and 2,600 ADMI, respectively. At the end of each treatment, aliquots of the decolorized samples were taken and centrifuged at 10,000 × *g* for 15 min. The final color concentration of the centrifuged supernatants of the treated samples was analyzed using a spectrophotometer (HACH 4000U) at 400 nm absorbance wavelength. All the experimental readings were triplicated in order to minimize bias. Finally, the percentage of color removal was calculated using Eq. 4;

(4) $$\%\mathrm{Color}\mathrm{removal}=\left(1-\frac{C_{e}}{C_{i}}\right)\times 100$$

where; *C*_*i*_ and *C*_*e*_ were color concentrations before and after treatment, respectively. The *C*_*e*_ and *C*_*i*_ were measured in ADMI scale. This scale is defined as American Dye Manufacturer’s Institutes (ADMI). Normally it uses a spectral method to calculate a single color value that is independent of hue. It is used for tinted effluents with colors that are different from the widely used Pt-Co units. ADMI scaling is program into the spectrophotometer (HACH 4000U) and it analysis the level of color concentration at absorbance wavelength of 400 nm, as mentioned earlier. The analysis of the color was done before and after each of the treatment.

### Performance Optimization

The Composite-Central-Design (Response Surface Methodology-Design Expert Version 10.0.7) was applied to develop the matrix for the optimization experiment with a total of 20 runs and the initial pH was fixed at ≈7 throughout. The multiple independent variables considered includes the *A*-inoculum size (5–25% v/v), *B-*initial color concentration (650–2,600, ADMI), and *C-*retention time (5–40 h). Basically, the interaction of the multiple’s variables with respect to the predicted-response (*Y*_*i*_-percentage of color removal) is usually expressed mathematically in the second-order polynomial model, as presented in Eq. 5;

(5) $$Y_{i}=\alpha_{0}+\sum_{i=1}^{k}\alpha_{i}\,x_{i}+\sum_{i=1}^{k}\alpha_{i\,i}\,x_{i}^{2}+\sum_{i=1}^{k}\sum_{j=1}^{k}\propto_{i\,j}x_{i}\,x_{j}$$

where, *Y*_*i*_ denotes predicted response, α_*o*_ represents an offset term, while *x*_*i*_ and *x*_*j*_ are the input variables. Also, α_*i*_ is *i*th linear-coefficient, α_*ii*_ is *i*th quadratic-coefficient and α_*ij*_ is represent *ij*th interaction-coefficient.

The experimental matrix based on the stated independent variables is summarized in Table 1. The optimization experiments were performed in a series of 250 mL Erlenmeyer flasks. Each flask contains a proportionate amount of POME concentration (650–2,600 ADMI) with inoculum (5–25% v/v) based on the developed experimental matrix to give 50 mL mixture of sample sizes. The inoculated mixtures were placed in an incubator shaker and treated at variable retention time (5–40 h) under steady agitation of 120 rpm. The treated samples were centrifuged and the color concentration of the supernatants was analyzed using a spectrophotometer. Each of the analysis was triplicated and then took the average.

**TABLE 1:** Optimization experimental layout summary for color removal.

Treatment factors	Unit	Symbol	Coded level		
			−1 (Lower level)	0	+1 (High level)
Inoculum size	%(v/v)	*A*	5	15	25
Initial color concentration	ADMI	*B*	650	1,625	2,600
Retention time	h	*C*	5	22.5	40

### Statistical Analysis

Analysis of variance was accomplished with the Design-Expert 10.0.7 software package. In order to minimize error, all the experimental readings and procedures were triplicated, and the average reading was obtained. The significance of the treatment factors was examined based on the magnitude of the *F*-values, while the *P* value less than 0.05 is considered to be statistically significant with 95% confidence level.

### Model Validation

The optimized treatment conditions were used to validate the regression model. The first three (3) predicted treatment conditions with the highest desirability index were selected and applied to examine the actual experimental percentage of color removal in the laboratory. The experimental data obtained were compared with the model predicted values based on the correlation coefficient (*R*^2^). In addition, a normal residual analysis was conducted to further verify the accuracy of the model.

### Analytical Methods

The analysis of the considered parameters (color, COD, turbidity, TSS, VSS, pH) were conducted in accordance with standard procedure (APHA, 2005). A calorimetric method was employed to determine the color, COD (vials of high concentration) and turbidity level using UV-spectrophotometer (HACH DR/4000U) at an absorbance wavelength of 400, 620, and 600 nm, respectively. Then, the percentage of color removal was determined using equation (4). The biomass suspended solids (TSS) were determined by filtering 100 mL of the sample through a 0.48 μm filter paper (Whatman, China); the retained residues were oven-dried at 105°C for 5 h. Whereas the VSS was determined by igniting the dried sample residue at 550°C using muffle furnace for a period of 15 min. The TSS and VSS were determined using Eqs 6 and 7, respectively. The pH was measured using a digital pH-meter (Ionix pH5S).

(6) $$\text{TSS},\left(\frac{\text{mg}}{\text{L}}\right)=\frac{M_{\text{solutes}}}{V_{s}}\times 1000$$

(7) VSS,(mgL)=Msolutes-M550⁢C∘Vs×1000

where; *M*_solute_ is mass of solute (mg); *M*_550 C_ is solute mass after ashed (mg); TSS is total suspended solid (mg/L) and VSS is volatile suspended solid (mg/L).

## Results and Discussion

### Physicochemical Characterizations

Table 2 shows POME’s initial physicochemical parameters. The concentrations of the analyzed parameters exceeded the DOE discharge limits. This shows that the color concentration and COD of the discharge is 2,600 ADMI and 1,264 mg/L, respectively. The contaminants concentration are far beyond the limits. Also, 320 mg/L of NH_3_-N alongside with the excessive concentration of suspended solids (TSS and VSS) were observed. However, only the pH (8.5) was within the acceptable DOE limit (Table 2). Therefore, discharging such partially treated wastewater is hazardous due to the high concentration of the carcinogenic colorants (Kamal et al., 2019). Nonetheless, the carbonaceous complex colorants present in POME could be utilized as a sole carbon source for the indigenous and/or inoculated bacteria under favorable conditions, thereby metabolizing it into simpler and less harmful compounds (Azman et al., 2019). This is the main focus of the current study.

**TABLE 2:** Comparison of initial physicochemical properties of the POME sample and discharge standard limit.

Parameter	Unit	Initial concentration	Doe limit*
Color	ADMI	2,600	100A
COD	mg/L	1,264	100
pH	–	8.5	5–9
TSS	mg/L	1,540	200
VSS	mg/L	470	200
Turbidity	FAU	1,870	50
NH_3_-N	mg/L	320	20

*Source: Din (2017).

### Viability Analysis of Inoculum

**FIGURE 2:**
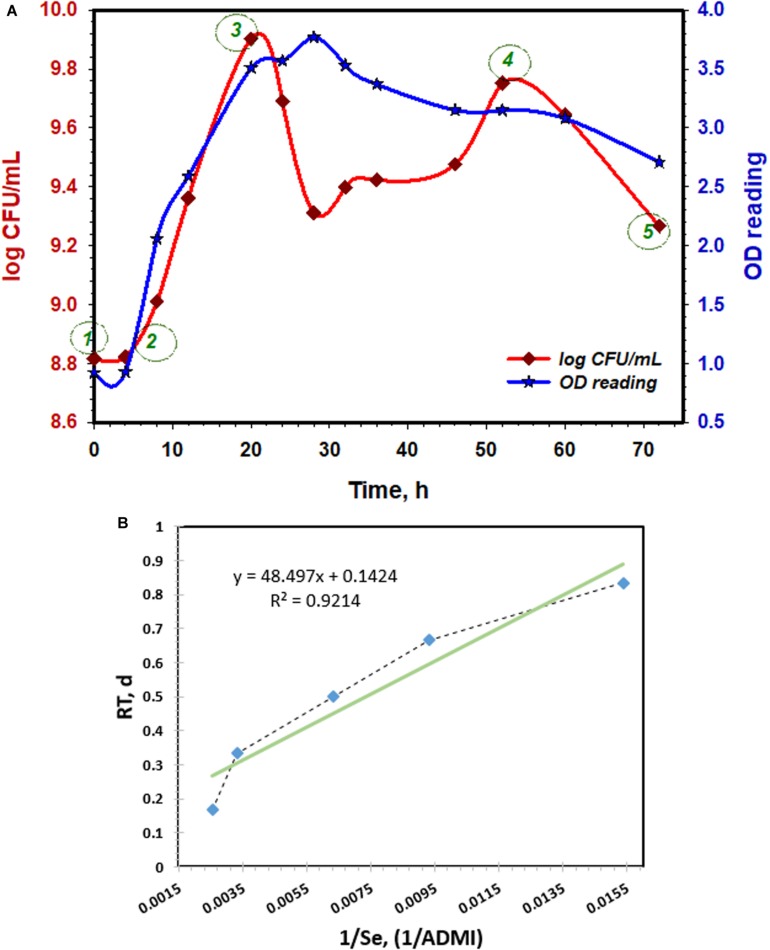
**(A)** Growth trend of *Klebsiella Pneumonia* ABZ11 in terms of log CFU/mL with respective OD reading at 600 nm for a total period of 72 h. **(B)** Monod Kinetic Model linearized plot fitted with experimental data of growth rate during the lag-exponential phase.

Figure 2A shows the coliform counts (log CFU/mL) with the corresponding OD readings at every growth phase in respect to retention time. As indicated in the figure, microbial growth between ① and ② presents the lag phase which lasted for ≈3.3 h and the corresponding OD readings were about ∼0.9. At this stage, the inoculum prepared for reproduction via binary fission and also synthesizes various inducible enzymes suitable for the subsequent degradation and metabolic processes (Cortes-Tolalpa et al., 2017). Therefore, a noticeable increase in the coliform is not expected at this phase (Oladipo et al., 2018). Conversely, a significant coliform reproduction takes place between ② and ③ such that an exponential increase in the population density from 8.8 to 9.9 log CFU/mL was achieved in this phase. This shows that a maximal rate of cells multiplication at minimal reproduction-time was attained. The maximum coliform (9.9 logs CFU/mL) was attained after 20 h retention with a corresponding OD reading of 3.5 (Figure 2A). At this exponential growth phase, the cellular constituents are produced at constant amounts relative to each other, thus showing the steadiest rate in the kinetic degradation process as well as the biochemical activities (Vello et al., 2018). On this note, the maximum growth yield, active metabolism and the highest colorant degradation efficiency are expected in this phase. Further extension in the retention-period between ③ and ④, a malignant and unsteady state in the viability of the cells were observed (Figure 2A). This might be due to the depletion in the available nutrient which is not adequate to sustain the densely populated microbes (Langemann et al., 2010). As a result, an endogenous process took place such that actives cells feed on one another. Thus, a dramatic reduction in the coliform count from 9.90 to 9.25 log CFU/mL. However, at retention period of 52 h, the coliform count appreciated to ∼9.7 log CFU/mL. This suggests that after the initial coliform depletion, the available limited biodegradable nutrients were adequate to sustain the biochemical activities. Afterward, a steady diminishing in the coliform count with respect to retention-time set-in between ④ and ⑤. This observation prevailed because the number of dying cells exceeds that of the new-born cells at this stage, thereby amounting to a dramatic diminution in the viable bacterial cells (Figure 2A). This stage indicates the death phase of the growth analysis.

The kinetic coefficients (μ*_max_* and *K*_*s*_) of the bacteria growth were determined based on color depletion during the lag-exponential growth phase (Park et al., 2015). Throughout, the magnitude of color depletion was measured in ADMI. Figure 2B shows the linearized plot of the model such that the gradient (*K*_*s*_/μ_max_) and intercepts (*1/*μ_max_) were used to determine the coefficients. Initially, the best line of fits was obtained using the least square of the linear regression method and the corresponding values for each coefficient were calculated. The value of μ_max_ and *K*_*s*_ within the considered growth phase was 7.022 d^–1^ and 340.63 ADMI d^–1^, respectively. Thus, the modified kinetic model based on the determined coefficients is expressed in Eq. 8;

(8) $$\frac{1}{\text{RT}}=\frac{7.022\times C_{e}}{340.63+C_{e}}$$

where; *C*_*e*_ is the remnants concentration of the treated POME (ADMI); while RT is the treatment retention time, (*d*).

Despite the recalcitrant nature of the colorants present in POME, the μ_max_ and *K*_*s*_ obtained in this study were comparably superior to that reported by Zhang et al. (2017), Ebrahimi et al. (2016) and Gonçalves et al. (2016). Collectively, the μ_max_ values stated in these works of literature ranges between 0.33 and 0.954 d^–1^, while *K*_*s*_ varies within 120–243.564 mg/L, which are considerably lower compared to values achieved in the present study (7.022 d^–1^ and 340.63 ADMI d^–1^). The higher value of μ_max_ (7.022 d^–1^) obtained is an indication of proliferating performance of the *Klebsiella Pneumonia* ABZ11, as well as its good adaption to the treatment conditions applied. Consequently, efficient microbial extracellular activities alongside with high biodegradation capacity are expected (Chan et al., 2010). In addition, *K*_*s*_ value is an index measuring the capacity of substrate (colorants) degradation (Fonseca et al., 2018). In this study, the *K*_*s*_ value was 340.63 ADMI d^–1^ as against the reported data in the literature (Ebrahimi et al., 2016; Gonçalves et al., 2016; Zhang et al., 2017). However, the excellent degradation capacity might be due to the intrinsic metabolic characteristic of the inoculum, which enables it to adapt easily to any change in the environment or treatment conditions, and thereby generating suitable metabolic enzymes to sustain swift degradation of the colorants (Hameed and Ismail, 2018).

### Batch Decolorization Performance

#### Effect of Initial Concentration at Varied Treatment Time With a Fixed Inoculum Size of 15% (v/v)

**FIGURE 3:**
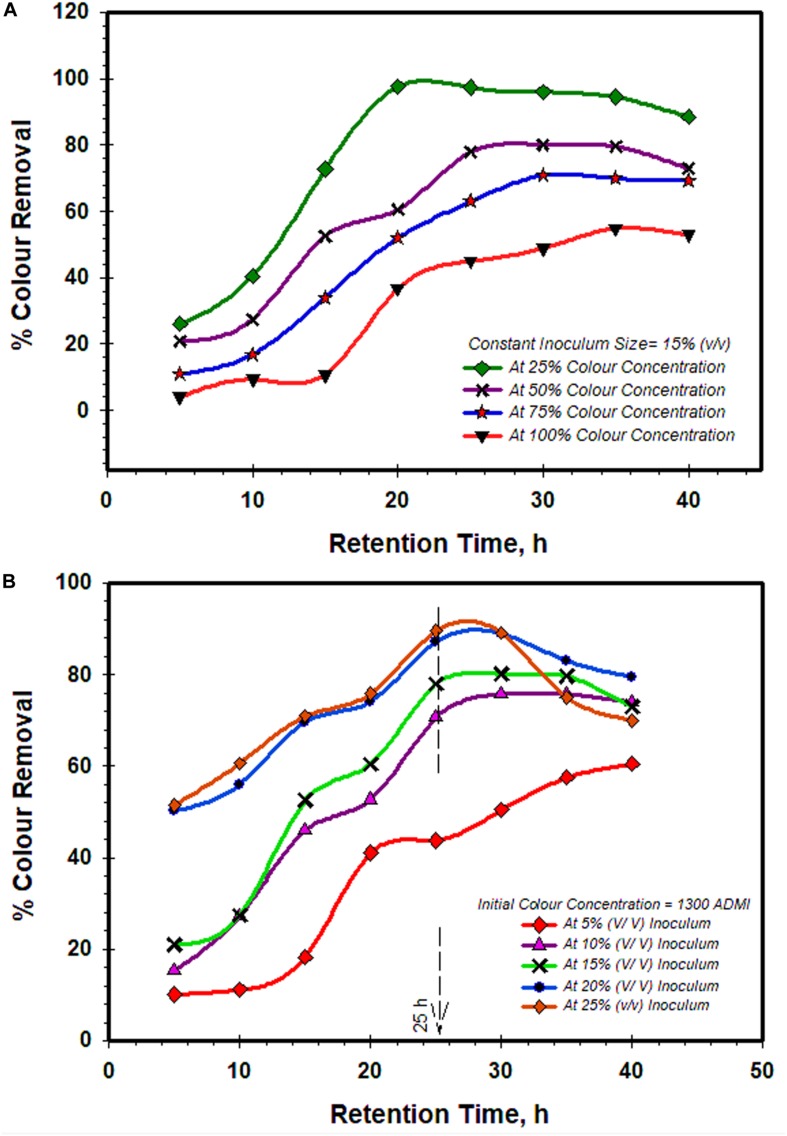
The combined effect of treatment retention time with **(A)** initial color concentrations, and **(B)** inoculum sizes on bio-decolorization performance by *Klebsiella Pneumonia* ABZ11.

The initial color concentration demonstrated considerable influences on the rate of biodegradation of the colorants (Figure 3A). At a lower initial color concentration of 650 ADMI, the biodegradation process was considerably faster with 15% (v/v) inoculum, and over 97.73% color removal efficiency was attained after 20 h retention time. The percentage of the color removal remains relatively steady but diminished to 86% at a longer retention time of 40 h. The reduction in the removal efficiency might be due to endogenous process resulted due to the depleted organic contaminants (colorants) and available dissolved oxygen (Park et al., 2015). At a higher color concentration of 2,600 ADMI, the decolorization was rather lagging and no any significant color removal noticed in the first 15 h of retention (Figure 3A). However, over 57% of color removal was achieved after a retention period of 35 h. The impeded decolorization process observed earlier at this initial concentration (2,600 ADMI) could be due to the predominant complex aromatic ring structure of the recalcitrant contaminants (such as colorants) which are not easily broken by the microbes’ metabolic and enzymatic activities (Hameed and Ismail, 2018). In addition, the presence of other toxic compounds (such as sulphonic groups) exerts an inhibitory effect on the extracellular activities of the microbes (Luo et al., 2014; Pernin et al., 2018). Thus, these compounds antagonize the overall bio-decolorization processes (Pernin et al., 2018). Nevertheless, the appreciable decolorization performance (57%) recorded afterward is an indication that the microbes were able to adapt and then secret suitable enzymes to degrade the high initial concentration (2,600 ADMI). In overview, it can be deduced that the efficiency of color removal decreases with increase in initial color concentrations, while the treatment retention time became much longer (Figure 3A). Researchers have attributed this effect to the excessive presence of toxics-recalcitrant contaminants (such as colorants) which rather inhibits the metabolic and physiological activities as well as the population density of the microbes (Luo et al., 2014; Hameed and Ismail, 2018; Pernin et al., 2018). Thus, the resulted relegation in the overall bio-degradation performance of the inoculum. This remark is also in accordance with other similar studies (Luo et al., 2014; Hameed and Ismail, 2018; Pernin et al., 2018).

#### Effect of Inoculum Sizes at Varied Treatment Times With a Fixed Initial Color Concentration of 1,300 ADMI

Figure 3B shows the effect of inoculum sizes with respect to treatment time on the color removal performance. A fixed initial color concentration of 1,300 ADMI was applied while the inoculum size was varied from 5 to 25% (v/v) with respect to treatment retention time (5–40 h). At inoculum size of 5% (v/v), a protracting lagging in the decolorization process (∼10.15%) was observed during the first 10 h of treatment time (Figure 3B). After 25 h of retention, an appreciable increase in the decolorization efficiency of 44% was attained and then improved to 60% after 40 h. However, it was generally noticed that higher inoculum size gives better decolorization performance. For example, at the 25 h of retention time, the treatment with inoculum sizes of 10, 15, 20, and 25% (v/v) recorded 70, 78, 87.5, and 89.25% of color removal. This advocate that with higher inoculum size, adequate active sites in conjunction with the intensive synergy of the metabolic process of the dense microbes are available to effectively degrade the colorants (Kumaran et al., 2016). Though, it can be perceived from this figure that the 25% (v/v) inoculum size makes no conspicuous distinction in decolorization performance compared with that of 20% (v/v). Therefore, it is reasonable to deduce that beyond inoculum size of 20% (v/v), there is no proportionate increase in the bio-decolorization proficiency with the upsurge in the inoculum size. More so, at a longer retention time of 40 h, a dramatic reduction in the percentage of color removal (68%) was observed in the treatment with 25% (v/v) inoculum size. This might be due to the copious population density of the microbes with limited nutrients (biodegradable contaminants) and dissolved oxygen (Park et al., 2015). Thus, an endogenous process prevails and this consequentially undermines the overall bio-decolorization performance. This observation was in good agreement with previous works (Moosvi et al., 2005; Mohana et al., 2007; Hameed and Ismail, 2018). Moosvi et al. (2005) reported that low count of available active microbes can result from a protracted endogenous process which later relegates the normal secretion of enzymatic substances required for the biodegradation. Also, Hameed and Ismail (2018) validated that better decolorization performances were achieved with higher inoculum size but diminished after an optimal size ranges. In another similar study reported by Mohana et al. (2007), the results confirmed that the highest decolorization and COD removal were obtained at 15% (v/v), but further increase resulted in no noticeable improvement in the remediation performance.

### Optimization Results

#### Analysis of Variance and Regression Model

Three significant experimental factors (A-inoculum size, B-initial color concentration and C-retention time) influencing decolorization of POME were optimized using center composite design (a component of RSM). The level of influences of the factors was analyzed using ANOVA, as summarized in Table 3. It was noticed that the regression model was statistically significant with an *F*-value of 13.46 and 0.0002 prob > *F* value. The *P*-value of 0.0002 implies that the model has less than 0.2% chance of bias due to noise. From the results, the level of significant modal terms was in the following order C, A and B with *F*-values of 48.57, 32.77, and 23.17, respectively. In addition, the terms of the polynomial such as C^2^ and A^2^ demonstrated considerable influences with modal *F*-value of 12.10 and 9.00, while B^2^ recorded the least (4.45). Based on the level of *F*-value, C exhibited the most significant influence on the bio-decolorization process and then A was observed next to it. Thus, only the significant factors (C, A, B, A^2^, C^2^ and B^2^) were used for developing the regression model Eq. 9. This is procedure is in accordance with Ghani et al. (2017).

**TABLE 3:** ANOVA for response surface quadratic model.

Source	Sum of Squares	*df*	Mean square	*F* value	*P*-value Prob > *F*	
Model	1,6907.93	9	1,878.66	13.46	0.0002	significant
A-Inoculum size	4,573.67	1	4,573.67	32.77	0.0002	–
B-Initial color concentration	3,234.80	1	3,234.80	23.17	0.0007	–
C-Retention time	6,780.16	1	6,780.16	48.57	<0.0001	–
AB	15.82	1	15.82	0.11	0.7433	–
AC	255.95	1	255.95	1.83	0.2055	–
BC	239.26	1	239.26	1.71	0.2197	–
A^2^	1,256.55	1	1,256.55	9.00	0.0133	–
B^2^	342.28	1	342.28	4.45	0.0384	–
C^2^	1,688.73	1	1,688.73	12.10	0.0059	–
Residual	1,395.86	10	139.59	–	–	–
Lack of fit	1,392.57	5	278.51	–	–	–
Pure error	3.28	5	0.66	–	–	–
Cor total	18,303.78	19	–	–	–	–
*R*^2^	0.9237	–	–	–	–	–
Adjusted-*R*^2^	0.8551	–	–	–	–	–

$$\%\mathrm{Color}\mathrm{removal}=5.4439+4.57547A-4.10414\times 10^{-3}$$

(9) $$B+2.27197\,C-0.10516\,A^{2}-5.16608\times 10^{-6}\,B^{2}-0.04228\,C^{2}$$

**FIGURE 4:**
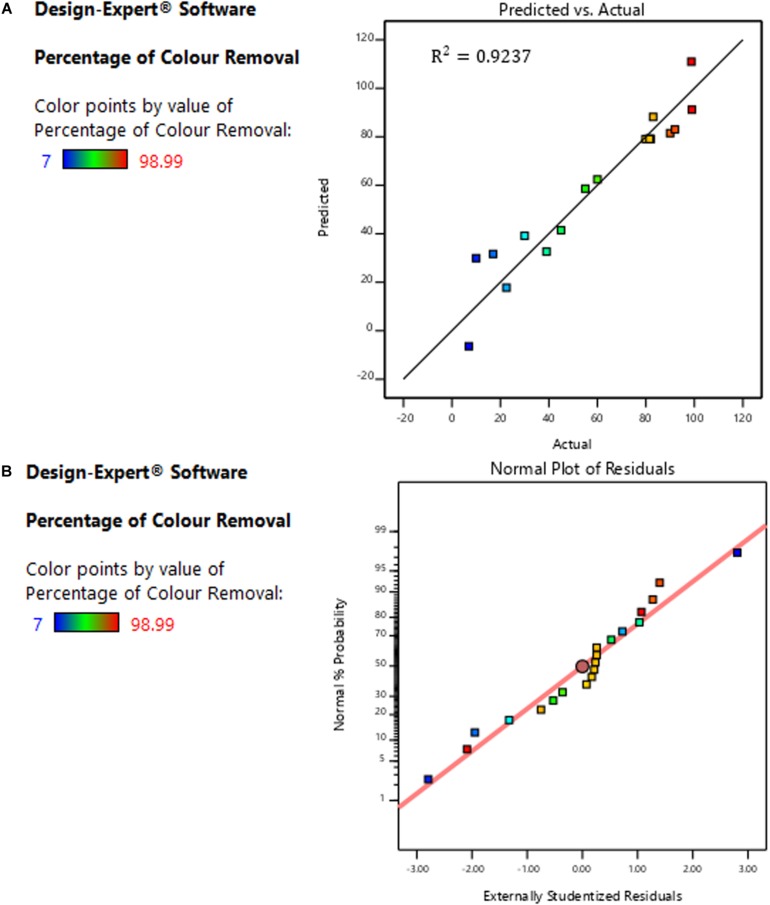
**(A)** Comparison between model-predicted and actual experimental value. **(B)** Normal residuals plots showing level deviation from diagonal points.

Figure 4A exemplifies the model predicted and actual values for the decolorization percentage. A good agreement was noticed between the predicted and actual values with 0.9237correlation (*R*^2^), and also the deviation from diagonal points was insignificant. Fundamentally, the 0.9237 *R*^2^ indicates that the notable factors (C, A, B, A^2^, C^2^ and B^2^) have over 92% impacts on the bio-decolorization process (Figure 4A). Furthermore, the high *R*^2^ (0.9237) value obtained demonstrate the reliability and confidence of the regression model, and thus it may be employed to navigate the design-space (Ghani et al., 2017). The accuracy of the model was further analyzed using normal plots of residual for the percentage of color removal, as presented in Figure 4B. Basically, the normal residuals point-out the amount at which the developed model satisfies ANOVA assumptions, while the studentized residuals is an index showing the level of deviation between the predicted and actual values (Mishra et al., 2019). From Figure 4B, it can be observed that virtually all the data points were on a straight diagonal line with insignificant deviations and this is an indication that application of response transformation is not required (Mishra et al., 2019). Therefore, it can be reasonably deduced that no apparent bias with the normality.

#### Perturbation Analysis

**FIGURE 5:**
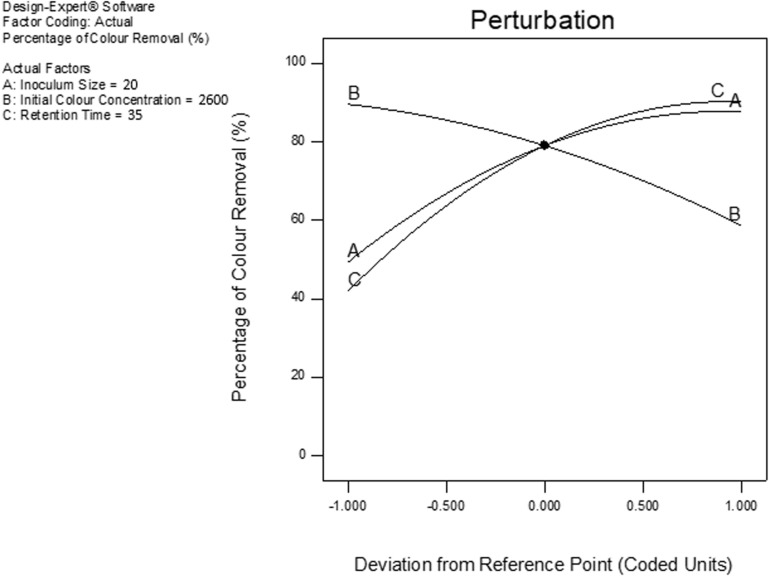
Perturbation plot showing the common point and influence of factors on the percentage of color removal.

The combined effect of the three treatment factors (A, B and C) at a common point of retort was examined by the statistical design based on perturbation plot (Taherdanak et al., 2015). From Figure 5, the thinness of the curve reflects how subtle the response was whenever there are vicissitudes in any of the treatment factors (Mishra et al., 2019). It is obvious that the inoculum size (A), initial color concentration (B) and the retention time (C) have a considerable influence on the percentage of color removal. The optimal percentage of color removal of 81.5% was attained at the inoculum size of 20% (v/v) and the retention period of 35 h with an initial color concentration of 2,600 ADMI (without dilution). Both A and C demonstrated a positive correlation with the percentage of color removal, while B exhibited a negative correlation, (Figure 5). Essentially, the perturbation figure shows the common point for the combined factors where the highest synergistic influences were attained to sustain optimal decolorization process by the *Klebsiella Pneumonia* ABZ11. This implies that at this common perturbation point, the effect of the antagonist factors (AB, BC, and AC) are minimal (Eltarahony et al., 2019; Mishra et al., 2019). Hence, the optimal treatment conditions at this point sustain the *Klebsiella Pneumonia* ABZ11 for effectual proliferation, metabolic activities alongside with protonic-balance which resulted in better degradation of the contaminants, and also neutralization of the toxic colorants (Mishra et al., 2019).

#### Optimized Synergy Effect of Inoculum Size and Initial Color Concentration

**FIGURE 6:**
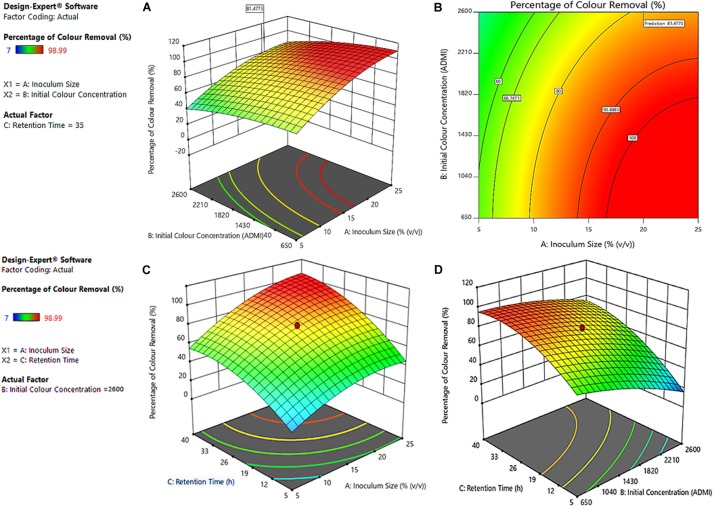
**(A)** Surface response, and **(B)** contour plot of the optimized synergistic effect of inoculum size and initial color concentration. The surface response of the combined effect of **(C)** Inoculum sizes-A and Treatment retention Time-C, and **(D)** Initial color Concentrations-B and Treatment retention Time-C on percentage of color removal.

The optimized response surface and contour plot for the combined effect of factors A-inoculum sizes and B-initial color concentration on the percentage of color removal are presented in Figure 6A and (B), respectively. From Figure 6A, it can be noticed that the percentage of color removal increases with A but decreases with B. At the highest initial color concentration (B) of 2,600 ADMI (without dilution) and 20% (v/v) inoculum size, the optimal bio-decolorization of 81.4773% was attained after 35 h retention, (Figure 6A). It can be detected from Figure 6B that lower color concentrations (650 ADMI) give better performance (65–98.90%) irrespective of the inoculum sizes (A) and retention times (C). The negative correlation of initial color concentration with the remediation performance has been attributed to the predomination of toxic-recalcitrant contaminants characterized by complex structure (Neoh et al., 2017). Thus, the complexity of recalcitrant contaminants rather inhibits the extracellular activities of the bacteria thereby retarding the rate of decolorization (Hameed and Ismail, 2018).

#### Optimized Synergy Effect of A-Inoculum Size and C-Treatment Retention Time

Figure 6C shows the surface response 3-D plot of synergistic influences of the A-inoculum sizes with respect to C-treatment retention time. It is obvious that both the retention time (C) and the size of inoculum (A) have a positive correlation with the percentage of color removal. From Figure 6A, the lowest percentage of color removal was obtained with 5% (v/v) inoculum and 5 h retention time. However, a dramatic increase in the color removal was noticed with higher inoculum sizes (C). For example, at an optimal inoculum size of 20% (v/v) with a retention time of 35 h, over 81.47% of decolorization was attained even with the highest color concentration of 2,600 ADMI (without dilution). This implies that at the optimal condition, the effect of the antagonist factors (AB, AC and BC) were minimal while the metabolic activities of the microbes were maximized (Figure 5; (Mishra et al., 2019).

#### Optimized Synergy Effect of B-Initial Color Concentration and C-Treatment Retention Time

Figure 6D shows the surface response of the synergy effect of the A-initial color concentrations with respect to C-treatment retention times. The surface response curbed in the red region of the plot indicates excellent color removal efficiency (70–98%) and this was noticed at a lower initial concentration (650 ADMI). Conversely, at a higher initial color concentration (2,600 ADMI), a sudden decrease in the overall bio-decolorization efficiency was observed (Figure 6D). This might be due to the presence of excessive recalcitrant colorants which in turn inhibits the degradation process. Although, an appreciable increase in the decolorization efficiency of 81.4775% was attained at optimal retention time (35 h) and inoculum size of 20% (v/v). At this optimal treatment conditions, the common perturbation point with minimal effect of antagonist factors was established (Figure 5). Based on this observation, it can be deduced that at longer C, the microbes gradually adapt to the changes in the B and thereby developing suitable enzymes to facilitate degradation of the complex colorants (Chan et al., 2010). This observation is strongly in agreement with the previous studies (Sani and Banerjee, 1999; Islam et al., 2018). Sani and Banerjee (Sani and Banerjee, 1999) reported that lower initial concentration gives better remediation performance, especially when the inoculum size and the retention period are in optimal ranges. Similarly, Islam et al. (2018) reported that biodegradation performance of bacterial communities is often influenced by the concentration of medium because of the alteration in the pH, which could consequentially inhibit the bacterial growth as well as the metabolic and physiological activities of the inoculum. Thus, impeding the overall remediation performance (Islam et al., 2018).

### Model Evaluation and Validation

The developed empirical regression model was validated by comparing the predicted with actual experimental values. The optimized treatment factors based on the set-criteria was employed to validate the accuracy of the model (equation 9). The criteria employed in this research targeted to minimize the inoculum size (A), and then maximize both initial color concentration (A) and percentage of color removal at the shortest retention time (C). Based on this, the first three experimental solutions recommended by the model were picked and verified in the laboratory by comparing with actual experimental values. The results were as presented in Table 4. The average percentages of the predicted and experimental color removal were 81.5 and 80.5%, respectively. The two values were in good conformity with the minimal and insignificant disproportion of 1.078%.

**TABLE 4:** Experimental validation results of the regression model.

Inoculum size [%(v/v)]	Initial color concentration (ADMI)	Retention time (h)	Predicted value (%)	Desirability	Experimental value (%)
20.000	2,599.993∼2,600	35.000	81.477	0.949	79.89
20.000	2,586.721∼2,600	35.000	81.777	0.948	80.91
20.000	2,599.973∼2,600	34.852∼35.000	81.359	0.947	80.59
Average value			81.538		80.46
					

In addition, the deviation between the predicted and experimental values was statistically analyzed using *t*-test and normality test (Shapiro–Wilk), and the summary of the analysis is presented in Table 5. Basically, the *t*-test measures the significance of the deviation in the means (Kim, 2015) while the normality test analyzed the correlation of the data (Bhering, 2017). The *P*-value of the *t*-test was 0.030, which is less than 0.050. This shows that the deviations in the predicted and experimental values are statistically insignificant at 95% confidence level. Also, the passed remarks obtained from the normality test shows that the two sets of data match the design probable pattern with a normal distribution (Bhering, 2017). Based on this validation, it can be rationally deduced that the regression model predicted the actual experimental values with insignificant deviations.

**TABLE 5:** Summary of the *t*-Test and Normality analysis of the predicted and actual experimental values.

*t*-Test							
Data source	*N*	Missing	Mean	SD	SEM	*P*-value	*t*
Predicted value	3	0	81.538	0.216	0.124	0.030	3.297
Experimental Value	3	0	80.463	0.522	0.301		
Normality	0.945						
Power of performed test with alpha (α) = 0.050: 0.660							

**Normality analysis (Shapiro–Wilk)**							

**Source**	***W*-statistic**			***P*-value**			**Remark**

Predicted data	0.941			0.530			Passed
Experimental data	0.956			0.595			Passed

## Conclusion

*Klebsiella Pneumonia* ABZ11 was successfully applied to decolorize POME by utilizing the contained colorants as the sole carbon source to sustain its metabolic and physiological activities. The decolorization performance was examined at variable inoculum sizes, initial color concentrations and treatment retention times with fixed agitation of 120 rpm and initial pH of 7. Percentage of color removal at 650 ADMI initial concentration, 15% (v/v) inoculum and 25 h retention time was 97.73%, but at a higher initial color concentration of 2,600 ADMI, the color removal efficiency reduces considerably to 57% even with longer retention of 35 h. This might be due to the inhibitory effect of the colorants. In addition, at optimal conditions of 20% (v/v) inoculum, 2,600 ADMI initial color concentration and 35 h retention time, the color concentration reduces drastically by 80.43% and this result was in good correlation with the regression-model predicted value of 81.5%. The accuracy analysis based on normality test shows good matches with the probable normal distribution pattern (*R^2^* = 0.9237). In addition, the Monod model excellently described the bio-decolorization kinetic process and the calculated maximum growth (μ_*max*_) and half-saturation constant (*K*_*s*_) were 7.023 d^–1^ and 340.569 ADMI d^–1^, respectively.

## Data Availability Statement

The datasets analyzed in this study are publicly available. The partial 16S rRNA gene sequence of the isolate has been deposited to GenBank: http://blast.ncbi.nlm.nih.gov/Blast.cgi, with the accession number KX266892.

## Author Contributions

MA and HM developed the research conceptualization and methodology. MA secured the software, did the investigation, data collation, formal analysis and original draft preparation. MA, HM, ZA, and KY validated the results. HM, ZA, KY, and AI provided the required resources, review-editing, visualization and supervision. HM was responsible for the project administration. Funding acquisition was achieved by HM and MA.

## Conflict of Interest

The authors declare that the research was conducted in the absence of any commercial or financial relationships that could be construed as a potential conflict of interest.

## Footnotes

1 http://blast.ncbi.nlm.nih.gov/Blast.cgi
