# Supplementary material for: Decolorization of Palm Oil Mill Effluent by Klebsiella Pneumonia ABZ11: Remediation Efficacy and Statistical Optimization of Treatment Conditions
Source: Front Microbiol. 2020 May 13;11:675. doi: 10.3389/fmicb.2020.00675 (PMC7237724; doi:10.3389/fmicb.2020.00675)
Supplement: Supplementary file 1 [file Data_Sheet_1.PDF]

Summary of Supplementary Information

a. Accession Number: KX266892
b. Access Link: http://blast.ncbi.nlm.nih.gov/Blast.cgi
c. A forward and reverse primer, (14-F: 5′- AGAGTTTGATCCTGGCTCAG-3′) and (1492-R: 5′- CGGTTACCTTGTTACGACTT -3′)
d. Figure S1 of Agarose gel electrophoresis showing genomic DNA isolated from ABZ11.

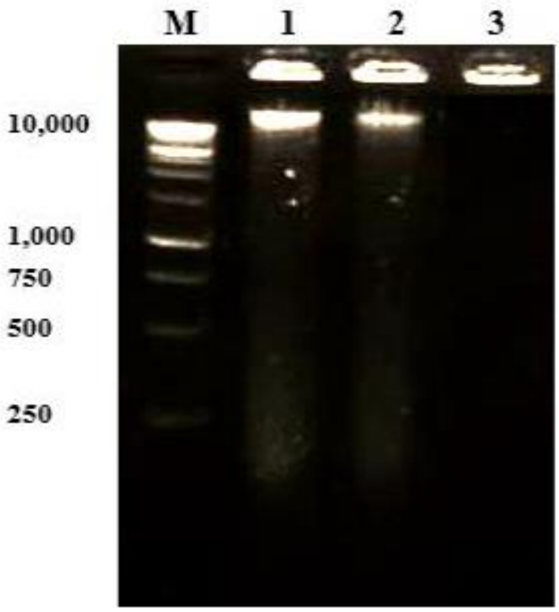

Figure S1

e. Figure S2 PCR amplification of the 16S rRNA gene of ABZ11showed the presence of 1.5 kb band.

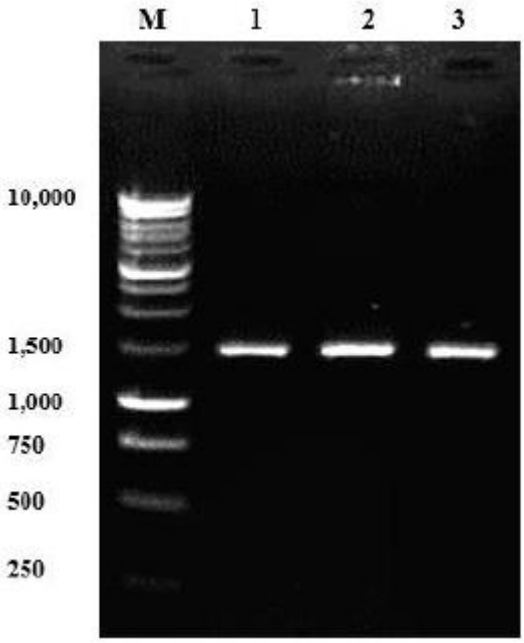

Figure S2

f. Figure S3 shows the partial 16S rRNA gene sequence of ABZ11, and it has been deposited to Genbank with the accession number KX266892

GTCGAGCGGTAGCACAGAGAGCTTGCTCTCGGGTGACGAGCGGCGGACGGGTGAGT
AATGTCTGGGAAACTGCCTGATGGAGGGGGATAACTACTGGAAACGGTAGCTAATA
CCGCATAACGTCGCAAGACCAAAGTGGGGGACCTTCGGGCCTCATGCCATCAGATGT
GCCCAGATGGGATTAGCTAGTAGGTGGGGTAACGGCTCACCTAGGCGACGATCCCTA
GCTGGTCTGAGAGGATGACCAGCCACACTGGAACTGAGACACGGTCCAGACTCCTAC
GGGAGGCAGCAGTGGGGAATATTGCACAATGGGCGCAAGCCTGATGCAGCCATGCC
GCGTGTGTGAAGAAGGCCTTCGGGTTGTAAAGCACTTTCAGCGGGGAGGAAGGCGG
TGAGGTTAATAACCTCATCGATTGACGTTACCCGCAGAAGAAGCACCGGCTAACTCC
GTGCCAGCAGCCGCGGTAATACGGAGGGTGCAAGCGTTAATCGGAATTACTGGGCG
TAAAGCGCACGCAGGCGGTCTGTCAAGTCGGATGTGAAATCCCCGGGCTCAACCTGG
GAACTGCATTCGAAACTGGCAGGCTAGAGTCTTGTAGAGGGGGGTAGAATTCCAGGT
GTAGCGGTGAAATGCGTAGAGATCTGGAGGAATACCGGTGGCGAAGGCGGCCCCCT
GGACAAAGACTGACGCTCAGGTGCGAAAGCGTGGGGAGCAAACAGGATTAGATACC
CTGGTAGTCCACGCCGTAAACGATGTCGATTTGGAGGTTGTGCCCTTGAGGCGTGGC
TTCCGGAGCTAACGCGTTAAATCGACCGCCTGGGGAGTACGGCCGCAAGGTTAAAAC
TCAAATGAATTGACGGGGGCCCGCACAAGCGGTGGAGCATGTGGTTTAATTCGATGC
AACGCGAAGAACCTTACCTGGTCTTGACATCCACAGAACTTAGCAGAAATGCTTTGG
TGCCTTCGGGAACTGTAAAACAGGTGCTGCATGGCTGTCCTCACCTCCGGTTGGGAA
ATGTTGGGTTAAGTCCCGCAACGAACGCAACCCTTATCCTTTGTTGCCAGCGGTTAG
GCCGGGAACTCAAAGGAAACTGCCAGTGATAAACTGGAGGAAGGTGGGGATGACGT
CAAGTCATCATGGCCCTTACGACCAGGGCTACACACGTGCTACAATGGCATATACAA
AGAGAAGCGACCTCGCGAGAGCAAGCGGACCTCATAAAGTATGTCGTAGTCCGGAT
TGGAGTCTGCAACTCGACTCCATGAAGTCGGAATCGCTAGTAATCGTAGATCAGAAT
GCTACGGTGAATACGTTCCCGGGCCTTGTACACACCGCCCGTCACACCATGGGTGG
GTTGCAAAAGAAGTAGGTAGCTTAACCTTCGGGAGGGCGCTACC

Figure S3

g. Figure S4 shows the phylogenetic relationship of *Klebsiella* sp. ABZ11 with the closely-related strains

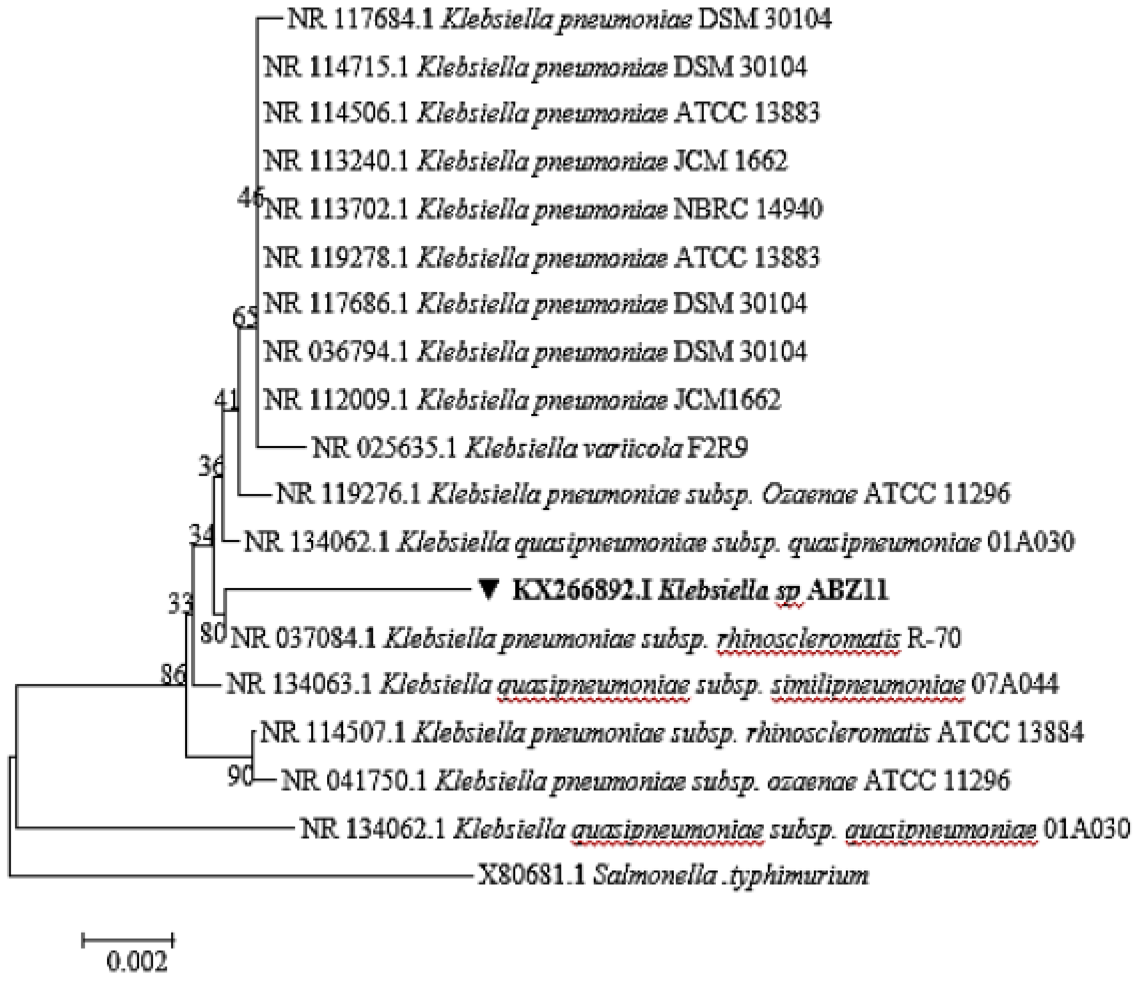

Figure S4
